# MRI brain structural and functional networks changes in Parkinson disease with REM sleep behavior disorders

**DOI:** 10.3389/fnagi.2024.1364727

**Published:** 2024-03-15

**Authors:** Fang Wang, Zhigang Zhu, Chuanbin Zhou, Yongyun Zhu, Yangfan Zhu, Chunyu Liang, Jieyu Chen, Bin Liu, Hui Ren, Xinglong Yang

**Affiliations:** ^1^Department of Neurology, First Affiliated Hospital of Kunming Medical University, Kunming, Yunnan, China; ^2^Department of Geriatrics, The Second Affiliated Hospital and Yuying Children’s Hospital of Wenzhou Medical University, Wenzhou, Zhejiang, China

**Keywords:** rapid eye movement sleep behavior disorder, Parkinson’s disease, functional connectivity, voxel-based morphometry, gray matter volumes

## Abstract

**Background:**

Rapid eye movement sleep behavior disorder (RBD) is common in individuals with Parkinson’s disease (PD). In spite of that, the precise mechanism underlying the pathophysiology of RBD among PD remains unclear.

**Objective:**

The aim of the present study was to analyze gray matter volumes (GMVs) as well as the changes of functional connectivity (FC) among PD patients with RBD (PD-RBD) by employing a combination of voxel-based morphometry (VBM) and FC methods.

**Methods:**

A total of 65 PD patients and 21 healthy control (HC) subjects were included in this study. VBM analyses were performed on all subjects. Subsequently, regions with significant different GMVs between PD patients with and without RBD (PD-nRBD) were selected for further analysis of FC. Correlations between altered GMVs and FC values with RBD scores were also investigated. Additionally, receiver operating characteristic (ROC) curves were employed for the evaluation of the predictive value of GMVs and FC in identifying RBD in PD.

**Results:**

PD-RBD patients exhibited lower GMVs in the left middle temporal gyrus (MTG) and bilateral cuneus. Furthermore, we observed higher FC between the left MTG and the right postcentral gyrus (PoCG), as well as lower FC between the bilateral cuneus (CUN) and the right middle frontal gyrus (MFG) among PD-RBD patients in contrast with PD-nRBD patients. Moreover, the GMVs of MTG (extending to the right PoCG) was positively correlated with RBD severity [as measured by REM Sleep Behavior Disorder Screening Questionnaire (RBDSQ) score]. Conversely, the FC value between the bilateral CUN and the right MTG in PD-RBD patients was negatively correlated with RBDSQ score.

**Conclusion:**

This study revealed the presence replace with GMV and FC changes among PD-RBD patients, which were closely linked to the severity of RBD symptoms. Furthermore, the combination of basic clinical characteristics, GMVs and FC values effectively predicted RBD for individuals with PD.

## Introduction

Parkinson’s disease (PD) ranks the second of the most common neurodegenerative diseases worldwide, primarily involving elderly individuals. It is featured by motor symptoms like bradykinesia, resting tremor, rigidity, as well as postural instability. However, PD is a complicated disorder, accompanied with numerous non-motor symptoms (NMSs), including constipation, cognitive impairment, chronic pain, anxiety, as well as rapid eye movement (REM) sleep behavior disorder (RBD)(Kalia and Lang, 2015; Zhou et al., 2022). RBD is distinguished by anomalous actions and the absence of muscle atonia in the REM sleep, thereby classifying it as a parasomnia. These behaviors include vocalizations, jerks, and motor movements, often associated with dream content related to REM sleep (Schenck and Mahowald, 2002).

Evidence suggests that idiopathic as well as symptomatic RBD are both closely linked to neurodegenerative diseases, particularly synucleinopathies like PD, multiple system atrophy (MSA), as well as dementia with Lewy bodies (DLB) (McCarter et al., 2012). However, the mechanisms underlying RBD symptoms remain controversial. Two hypotheses have been proposed: the cortical hypothesis together with the brainstem hypothesis, with a focus on the neocortex and limbic system (Iranzo, 2018). The ventral medial medulla (VMM) suppresses spinal in a state of typical REM sleep, thus restraining the motor cortex from triggering movements via spinal cord hyperpolarization. According to the cortical hypothesis, dysfunction of the VMM in RBD might result in the neocortex generating movements. Likewise, the VMM suppresses the red nucleus during regular REM sleep, thereby preventing movements in the spinal cord. Consequently, the brainstem hypothesis proposes that impaired function of the VMM in RBD permits excessive movements initiated by the red nucleus. Nonetheless, neither hypothesis encompasses the involvement of the limbic system. The findings by Guo et al. (2018) revealed elevated nodal measures within both the neocortex and limbic system, potentially eliciting activation of the ascending reticular activating system during REM sleep. This activation could induce a state akin to arousal, ultimately giving rise to atypical motor behaviors in individuals with PD and co-occurring REM behavior disorder (PD-RBD). Conversely, Li et al. (2017) presented evidence showcasing reduced activity of the primary motor cortex, suggesting compromised motor behavior regulation within REM sleep among PD-RBD patients. These contradictory outcomes and explanations highlight the pressing necessity for a more profound comprehension of the intricate mechanisms underlying RBD’s symptoms among PD patients.

As a highly effective approach for examining alterations in gray matter volume (GMV) with high spatial resolution (Ashburner and Friston, 2000), voxel-based morphometry (VBM) has been extensively employed to investigate structural changes within isolated brain regions among individuals with PD-RBD. Although studies have demonstrated modifications in functional connectivity (FC) in idiopathic RBD (Ellmore et al., 2013; Dayan and Browner, 2017), there has been limited understanding on FC among PD patients with RBD. Currently, investigation has few focused on the disparities in the structure combined with the function of PD patients based on the presence of RBD using either VBM or FC techniques. As a driving force behind the alternations of FC, GMV integrated with FC might offer enhanced understanding on relevant functional components (Hamilton, 1960; Goetz et al., 2008). The analysis of VBM combined with FC has been utilized across various diseases to gain further insights into the intricate substrate underlying these conditions (Tan et al., 2019; Wang et al., 2019; Zhao et al., 2020). Several previous studies have explored the structural and functional alterations in RBD and PD, the decrease of GM volume in PD-RBD patients was mainly concentrated in posterior brain regions, occipital, temporal and parietal (Jiang et al., 2021; Chen et al., 2022; Donzuso et al., 2024). Based on these findings, we hypothesized that PD-RBD patients exhibited specific changes in GMVs and FC in certain brain regions, which were closely associated with RBD. To test this assumption, our study was aimed to elucidate the neuroanatomical distinctions among PD patients with RBD from those without RBD (PD-nRBD). It was accomplished by analyzing volumetric dissimilarities in specific brain structures and exploring connectivity disparities from a comprehensive brain network standpoint. Furthermore, we conducted a comparative analysis of volumetric and connectivity parameters among the groups, and compared them to a control group consisting of healthy individuals (HC). Our results may contribute to the identification of imaging markers for PD-RBD and a deeper knowledge on the possible mechanisms.

## Methods

### Study population

We enrolled 65 patients with PD who were admitted and treated at the First Affiliated Hospital of Kunming Medical University between January 2022 and January 2023. PD diagnosis complied with the guidelines of the International Association for PD and Movement Disorders (Postuma et al., 2015). Controls were recruited from age-and sex-matched individuals without any other chronic conditions. Exclusion criteria included: (1) Parkinson’s syndrome or secondary PD; (2) Brain tumor and other neuropsychiatric disorders; (3) A history of intracranial surgery; (4) Contraindications to magnetic resonance imaging (MRI); (5) left-handedness.

This study was approved by the Ethics Committee of the First Affiliated Hospital of Kunming Medical University (2019-L-46). All participants provided written informed consent for their anonymized clinical data to be analyzed and published for research purposes upon admission to our hospital.

### Clinical assessment

Baseline information on all participants was collected from electronic medical records and face-to-face interviews. For PD patients, demographic and clinical data, including age, sex, years of education, as well as levodopa-equivalent daily dose (LEDD), were collected. The following scales were evaluated when the PD patients were in the “on” state. Motor symptoms were evaluated utilizing the Hoehn and Yahr (H-Y) Scale (Hoehn and Yahr, 1967) as well as Unified PD Ranking Scale Part-III (UPDRS-III) (Goetz et al., 2008). Levels of depression and anxiety were assessed using the Hamilton Depression Scale (HAMD) (Hamilton, 1960) and the Hamilton Anxiety Scale (HAMA) (Hamilton, 1959). Cognitive function was determined based on the Mini-Mental State Examination (MMSE) (Folstein et al., 1975). REM Sleep Behavior Disorder Screening Questionnaire (RBDSQ) was utilized for the assessment of RBD. Clinically possible RBD would be considered with a RBDSQ score higher than 5, along with reports from the patient or their family regarding disturbing dream experiences and related behaviors occurring during sleep (Stiasny-Kolster et al., 2007).

### Image acquisition and preprocessing

Utilizing a 3.0 T MR system (Discovery 750w, GE Healthcare, United Sttaes) with an eight-channel head coil, MRI data were acquired. During the MRI head routine sequence, rs-fMRI, and 3D-T1WI scans, individuals were told to close eyes, keep awake, and avoid active thinking in the scanning. Parameters for 3D-T1WI data included: (1) repetition time (TR): 8.2 ms; (2) echo time (TE): 3.2 ms; (3) turn angle: 12°; (4) matrix: 256 × 256, (5) field-of-view: 256 mm × 256 mm; (6) slice thickness: 1 mm. The parameters for rs-fMRI were: (1) TR: 2000 ms, (2) TE: 35 ms, (3) slice thickness: 4.0 mm; (4) inter-slice space: 0; (5) matrix: 64 × 64; (6) field of view: 240 mm × 240 mm; (7) voxel size: 3 × 3 × 3 mm; (8) time points: 240.

Preprocessing of 3D-T1WI images was conducted using the VBM8 toolbox in SPM8. Segmentation and spatial normalization were performed, and images were segmented into three regions: gray matter (GM), white matter (WM), as well as cerebrospinal fluid (CSF). Selecting the improved DARTEL registration method provided in VBM8, images of all subjects were registered to the MNI spatial template. After extracting the normalized GMV maps, a Gaussian kernel with a full-width at half-maximum (FWHM) of 8 mm was applied to achieve smoothing.

Using RESTPlus and SPM12 in MATLAB (R2013b), the preprocessing of rs-fMRI data was achieved. First, we removed the first ten time points and corrected slice timing to to avoid potential artifacts caused by scanner calibration and subjects’ adaptation to scanning environment. Then we removed the images that motion exceeded 2 mm or rotation along any direction exceeded 2° for head positioning correction. We also normalized data to the Montreal Neurological Institute template (voxels measuring 3 × 3 × 3 mm) and used the isotropic Gaussian kernel for spatial smoothing with a half-width of 6 mm. The remaining steps were composed by linear drift correction (on the white matter, cerebrospinal fluid, and 24 head motion parameters), covariate removal, and bandpass filtering (0.01–0.08 Hz) to get rid of low-frequency drift along with high-frequency physiological noise.

The regions of interest (ROIs), which were defined as regions with changes in GMVs within the subjects’ brain regions, were utilized as seed regions for voxel-wise FC analysis. We calculated the Pearson correlation coefficient between the mean time series within ROIs and other brain regions, and used Fisher’s r-to-z transformation to convert the resulting connectivity plot to a Z-plot.

### Statistical analysis

SPSS 26.0 software (IBM, Armonk, NY, United States) was adopted for statistical analysis. Categorical variables were described with proportions and assessed utilizing the chi-squared test. Besides, continuous variables with normally distribution were presented as mean ± standard deviation (SD) and compared utilizing two-sample t-tests as well as analysis of variance (ANOVA). In addition, continuous variables with skewed distribution were presented with median and interquartile range (IQR), and compared utilizing the Mann–Whitney U and Kruskal-Wallis tests.

The comparison of GMVs between groups were performed using a two-sample t-test in SPM12. To further minimize the impact of external factors, sex, age, years of education, UPDRS-III, MMSE, HAMD, and HAMA score were included as covariates in comparing PD-RBD and PD-nRBD before PSM. The covariates included in the other groups before PSM and the group after PSM were sex, age, and years of education. The corrected *p*-value utilizing the Gaussian random field (GRF) method was set at voxel *p* value less than 0.01 as well as cluster p value less than 0.05 (two-tailed). Clusters exhibiting differences with statistical significance within GMVs between groups were selected as regions of interest (ROI). Next, a two-sample *t*-test of FC with ROI between groups was performed in SPM12, including covariates such as sex, age, years of education (*p* < 0.001, uncorrected). GMVs and FC values with differences when comparing PD-RBD to PD-nRBD were extracted, and Spearman correlation was used to calculate the correlation with RBDSQ score. Additionally, receiver operating characteristic (ROC) curves were employed for the assessment of the predictive role of GMVs in discriminating between PD-RBD and PD-nRBD.

## Results

### Demographic and clinical characteristics of study patients

Table 1 presents the baseline demographic characteristics. No differences with statistical significance were found in sex, age, and education level among the HCs, and PD patients with or without RBD. No differences in LEDD and MMSE scores with statistical significance were observed between the PD groups based on the presence of RBD. However, in contrast with those without RBD, PD patients with RBD had relatively higher scores for H-Y, UPDRS-III, HAMD, and HAMA (Table 1).

**Table 1:** Clinical and demographic characteristics of study participants.

Characteristic	HC (*n* = 21)	PD-nRBD (*n* = 44)	PD-RBD (*n* = 21)	*p* value
Male	9 (42.9%)	24 (54.5%)	14 (66.7%)	0.301
Age (years)	64.3 ± 10.2	62.3 ± 11.3	66.9 ± 7.2	0.239
Education (years)	6 (6–9)	8 (6–12)	9 (6–14)	0.631
H-Y stage	NA	2 (1–3)	3 (2–3)	0.008*
LEDD (mg)	NA	337.5 (187.5–531.3)	475.0 (302.5–608.0)	0.084
UPDRS-III	NA	27.1 ± 15.1	37.1 ± 15.7	0.019*
MMSE	NA	25.0 (19.0–28.0)	23.0 (17.5–26.5)	0.216
HAMD	NA	12.1 ± 10.2	19.5 ± 7.8	0.002*
HAMA	NA	15.4 ± 13.2	23.8 ± 9.8	0.006*

*p < 0.05. Values are presented in n (%), median (IQR) or mean ± SD, unless otherwise noted. Abbreviations: HC, healthy controls; PD-RBD, Parkinson’s disease with RBD; PD-nRBD, Parkinson’s disease without RBD; HAMA, Hamilton Anxiety Scale; HAMD, Hamilton Depression Scale; H-Y stage, Hoehn-Yahr stage; LEDD, levodopa-equivalent daily doses; MMSE, Mini-Mental State Examination; UPDRS-III, Unified Parkinson’s Disease Rating Scale Part-III.

### Alteration in GMVs among PD patients with RBD

**Figure 1:**
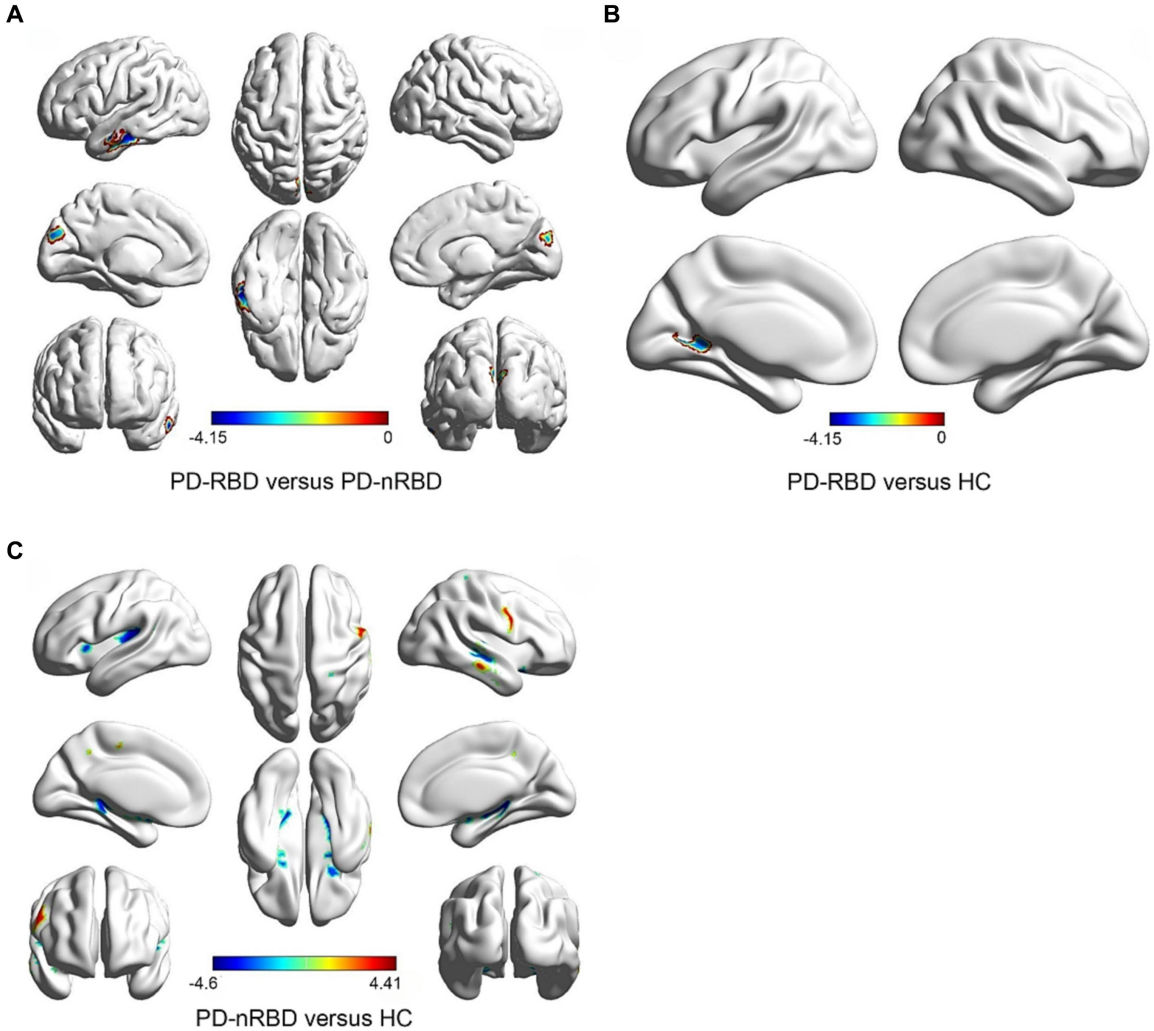
Comparison of GMVs in groups. In contrast with those without RBD, PD patients with RBD showed lower GMVs in MTG. L, CUN.L, CUN.R. Covariables are gender, age, education, H-Y, UPDRSIII, HAMD, and HAMA **(A)**; Compared to HCs, PD-RBD patients showed the GMVs lower in CAL. L, Covariates are gender, age and education **(B)**; Compared to HCs, PD-nRBD patients showed the GMVs lower in MTG. R, PUT.L, PUT. R, HIP.L, STG. L, PoCG.R and higher in the MTG.R, PreCG. R, MCG.L, MCG.R, Covariates are gender, age and education **(C)**. GMV, gray matter volume; PD-RBD, Parkinson’s disease with RBD; PD-nRBD, Parkinson’s disease without RBD; MTG, Middle temporal gyrus; CUN, cuneus; HC, Healthy controls; CAL, calcarine; PUT, putamen; HIP, Hippocampus; STG, Superior temporal gyrus; PoCG, Postcentral gyrus; PreCG, precentral gyrus; MCG, middle cingulum gyrus; L, left hemisphere; R, right hemisphere.

Lower GMVs within the left middle temporal gyrus (MTG) and bilateral cuneus (CUN) were found in PD patients with RBD than those without RBD (Table 2; Figure 1A). PD-RBD showed lower GMVs in the left calcarine (CAL) than the HC, (Table 2; Figure 1B). On the other hand, PD-nRBD showed lower GMVs in the right MTG, bilateral putamen (PUT), left hippocampus (HIP), left superior temporal gyrus (STG), as well as right postcentral gyrus (PoCG) than the HC (Table 2; Figure 1C), while showing higher GMVs in the right MTG, right precentral gyrus (PreCG), and bilateral middle cingulum gyrus (MCG) (Table 2; Figure 1C).

**Table 2:** Comparison of Gray matter volume between groups.

	Regions	Number of voxels	Peak activation strength (T)	Peak coordinates		
				x	y	z
PD-RBD < PD-nRBD						
	Temporal_Mid_L	898	−4.1544	−55.5	−22.5	−21
	Cuneus_L, Cuneus_R	375	−3.9194	4.5	−88.5	25.5
PD-RBD < HC						
	Calcarine_L	234	−3.439	−22.5	−51.0	0
PD-nRBD > HC						
	Temporal_Mid_R	443	4.4134	64.5	−4.5	−19.5
	Precentral_R	307	3.8969	52.5	1.5	28.5
	Cingulum_Mid_R, Cingulum_Mid_L	370	3.5289	1.5	−34.5	48
PD-nRBD < HC						
	Temporal_Mid_R	448	−4.6033	51	−12	−24
	Putamen_R	734	−3.9001	24	−40.5	4.5
	Putamen_L	567	−3.9604	−19.5	12	0
	Hippocampus_L	228	−4.2342	−19.5	−37.5	6
	Temporal_Sup_L	850	−3.762	−39	−28.5	13.5
	Postcentral_R	259	−4.1035	28.5	−30	55.5

GM, Gray matter; HC, Healthy controls; PD-RBD, Parkinson’s disease with RBD; PD-nRBD, Parkinson’s disease without RBD; MTG, Middle temporal gyrus; CUN, cuneus; CAL, calcarine; PUT, putamen; HIP, Hippocampus; STG, Superior temporal gyrus; PoCG, Postcentral gyrus; PreCG, precentral gyrus; MCG, middle cingulum gyrus; L, left hemisphere; R, right hemisphere.

### Differences in the FC of PD-RBD and PD-nRBD

**Figure 2:**
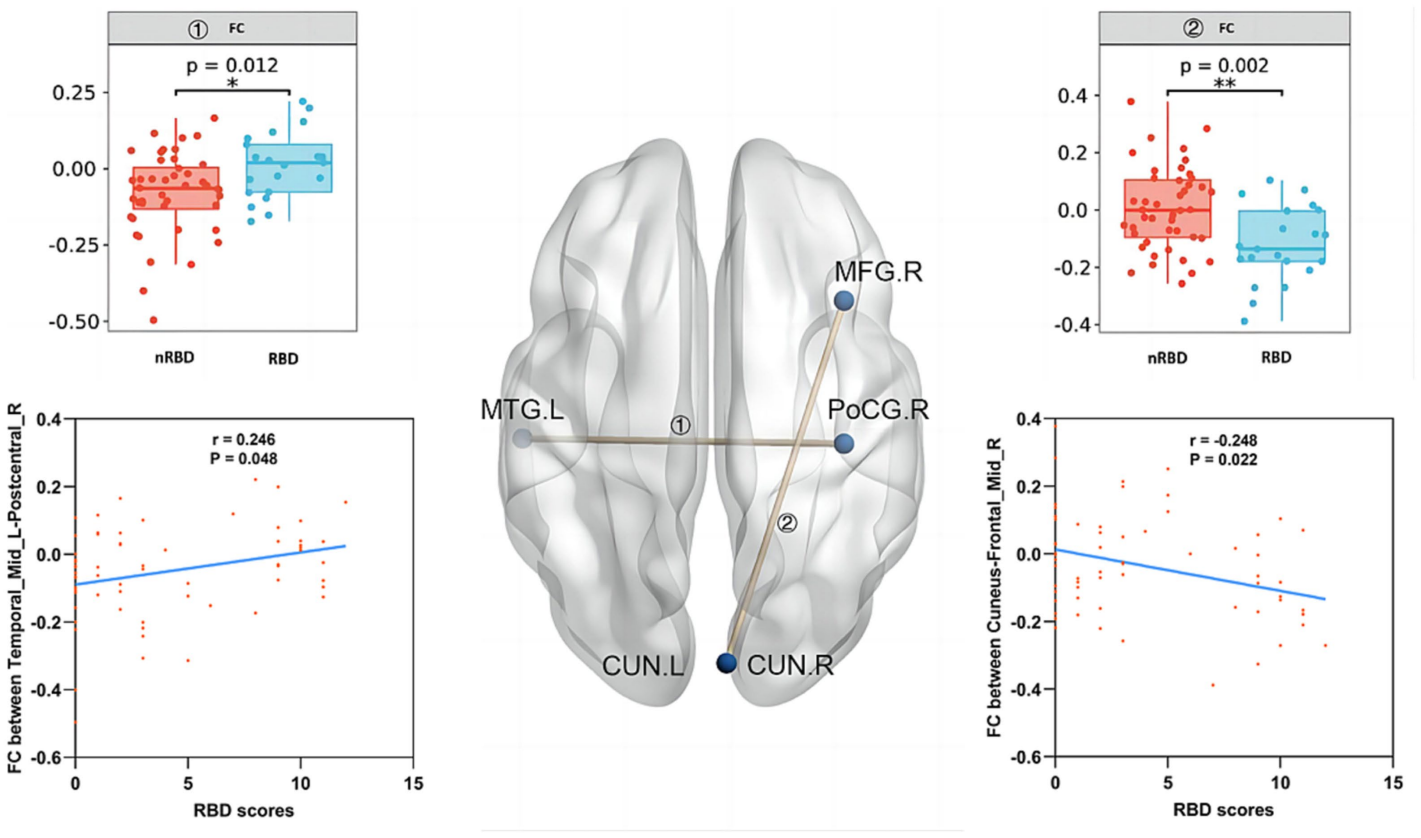
Compared to PD-nRBD, PD-RBD showed higher FC between left middle temporal gyrus (MTG) and right postcentral gyrus (PoCG), while lower FC between bilateral cuneus (CUN) and right middle frontal gyrus (MFG). The gray matter volumes (GMVs) of the left MTG and bilateral CUN in PD patients were not correlated with its RBD score. RBD score was positively linked to FC between the left MTG and right PoCG among PD patients. Conversely, the FC value of the bilateral CUN-right MFG among PD patients was negatively linked with its RBD score.

A two-sample t-test was conducted on FC with bilateral CUN and left MTG as ROI between PD-nRBD and PD-RBD, with covariates of age, years of education, as well as sex removed. Compared to those without RBD, PD-RBD demonstrated increased FC between the left MTG and right PoCG, while showing decreased FC between both sides of CUN and right middle frontal gyrus (MFG) (Table 3; Figure 2).

**Table 3:** Comparison of functional connectivity between PD-RBD and PD-nRBD.

ROI	Cluster	Cluster peak coordinates			Number of voxels	Peak activation strength (T)
		x	y	z		
Temporal_Mid_L	Postcentral_R	39	−24	33	27	−4.7492
Cuneus_L Cuneus_R	Frontal_Mid_R	39	18	27	20	4.0246

ROI, regions of interest; PD-RBD, Parkinson’s disease with RBD; PD-nRBD, Parkinson’s disease without RBD; MTG, Middle temporal gyrus; CUN, cuneus; L, left hemisphere; R, right hemisphere.

### Clinical correlation analysis

No correlation was found between GMVs of the left MTG and bilateral CUN in PD patients and their RBD scores (Figure 2). However, the RBD score was in a positive relationship with FC of the left MTG and right PoCG among PD patients (*p* = 0.048, *r* = 0.246). Conversely, the FC value of bilateral CUN-right MFG in PD patients showed a negative correlation with their RBD score (*p* = 0.022, *r* = −0.248) (Figure 2).

### Roc curves for the prediction of RBD in PD

**Figure 3:**
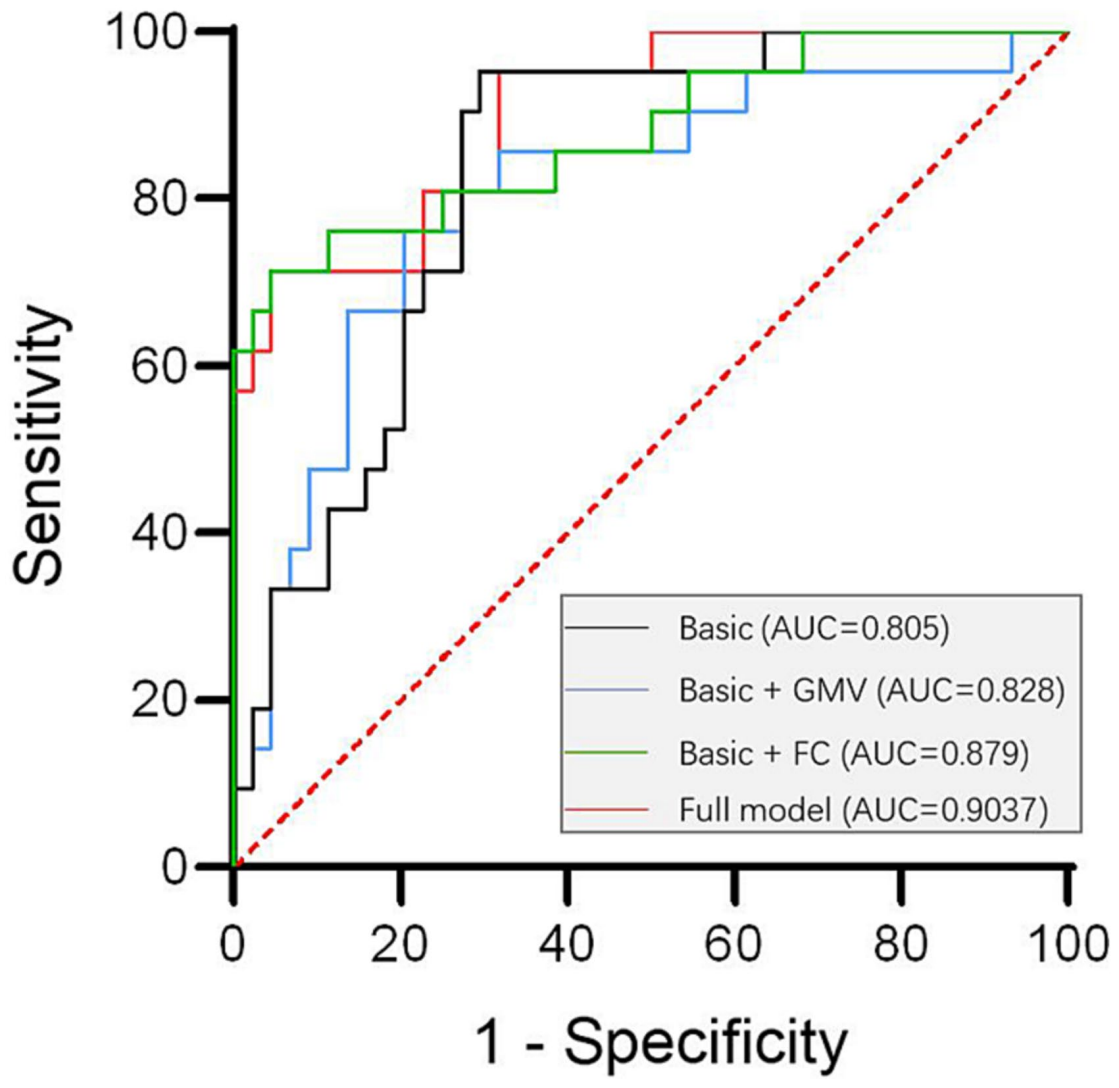
ROC curve analysis for the establishment of a diagnostic model for PD-RBD. The optimal specificity and sensitivity were yielded by using the full model combining basic clinical features (including age, education level, disease duration, H-Y, UPDRS-III, HAMD, HAMA, MMSE score) with the neuroimaging biomarkers: GMVs of left MTG and bilateral CUN, FC values between left MTG and right PoCG as wellas between bilateral CUN and right MFG (AUC = 0.9037, 95%CI 0.828–0.979, *p* < 0.001). FC, functional connectivity; GMV, gray matter volumes.

The diagnostic model for PD-RBD was evaluated using the ROC curve. By constructing a binary Logistic regression model for joint diagnosis, we computed the combined predictive probability of eight indicators encompassing age, education level, disease duration, H-Y stage, UPDRS-III, HAMD, HAMA and MMSE score. The ROC curve was plotted for Basic model using this predictive probability and the area under the curve was calculated. The Basic model serves as the foundation, upon which we incorporated GMVs to create the Basic + GMVs model, introduced FC to establish the Basic + FC model, and combined both GMVs and FC to form the full model. The accuracy of prediction increased from the basic model (AUC = 0.805, 95% CI 0.687–0.924, *p* < 0.001) to the model combining baseline features with GMVs of the left MTG and bilateral CUN (AUC = 0.828, 95% CI 0.728–0.928, *p* < 0.001). Further improvement in the accuracy of prediction was achieved by including FC values in the models combined with basic characteristics (AUC = 0.879, 95% CI 0.782–0.976, *p* < 0.001). In addition, the most optimal combination of specificity and sensitivity were yielded using the full model, incorporating basic clinical features (age, education level, duration of diseases, H-Y, UPDRS-III, HAMD, HAMA, MMSE score), neuroimaging biomarkers such as GMVs of the left MTG and bilateral CUN, as well as FC values between the left MTG and right PoCG, or between bilateral CUN and right MFG (AUC = 0.9037, 95% CI 0.828–0.979, *p* < 0.001) (Figure 3).

## Discussion

In the present study, we observed lower GMVs in the left MTG and bilateral CUN in PD patients accompanied with RBD, elevated FC between the left MTG and right PoCG, along with decreased FC between bilateral CUN and right MFG in contrast with those without RBD. By combining clinical and imaging data, we established an multiple dimensions diagnostic model for PD-RBD.

The MTG is located in the middle of the temporal cortex and is linked to sophisticated cognitive functions like intricate object visualization, meaningful interpretation through semantic processing, as well as socioemotional processing (Herlin et al., 2021). Given the violent behaviors and associated negative emotions in RBD, it is reasonable to speculate that the paralimbic cortex, involved in emotional control, is pivotal for the pathogenesis of PD-RBD (Dauvilliers et al., 2018). Previous researches using VBM demonstrated a smaller volume in the posterior brain, such as the temporal/parietal lobes, among PD patients with RBD confirmed by polysomnography (PSG) (Kim et al., 2016; Lim et al., 2016; Rahayel et al., 2019) or those with probable RBD (Ford et al., 2013) in contrast with those without RBD. Furthermore, PD patients accompanied with RBD displayed cortical thinning within either the inferior or superior regions of temporal cortex (Rahayel et al., 2019). Another MRI study showed extensive atrophy in the inferior temporal cortex, bilateral superior frontal gyri, along with left rostral middle frontal cortex among PD-RBD patients in contrast with idiopathic RBD (iRBD) patients, indicating significant paralimbic/limbic cortical changes (Pereira et al., 2019). Our findings partially supported these previous studies, suggesting the roles of the neocortex as well as the paralimbic system in the development of PD-RBD.

Furthermore, we found increased FC between the left MTG and right PoCG among PD-RBD patients. The right PoCG is a dominant region of the somatic sensation cortex, involved in motor-sensory feedback and neural pathways (Cappelletti et al., 2010; Fink et al., 2014). It is possible that the motor control and regulatory mechanisms exhibit compensatory responses to counterbalance the functional decline observed in the right postcentral gyrus of the somatosensory cortex within the sensorimotor network, thus preserving the overall circuitry integrity within the somatosensory-motor neural system (Wang et al., 2021). Increased FC between the left MTG and right PoCG may reflect compensatory mechanisms for sensorimotor integration deficits induced by other areas, maintaining the integrity of somatosensory-motor circuits (Ford et al., 2013).

In terms of the bilateral CUN, we observed lower GMVs in PD-RBD patients. The medial aspect of the occipital lobe is divided into CUN and lingual gyrus by the calcarine fissure, and the visual center is located in the occipital lobe, specifically in the cortex surrounding the calcarine fissure. The CUN, precuneus, as well as other regions within the posteromedial region of the parietal lobe are related with a wide range of complex tasks such as visualizing spatial images, retrieving memories, focusing attention, processing self-related information, and sleeping (Cavanna and Trimble, 2006). The occipital regions, which are responsible for visual processing in the brain are often affected in RBD patients. As a result, they may experience vivid and disturbing nightmares that involve visual hallucinations during sleep (Wang et al., 2010). Research indicates that there is a strong connection between the visual regions and the underlying physiological processes of RBD. According to an electroencephalography study, the visual areas in the brain, which are located above the primary visual cortex, may become active during REM sleep. This activation may then lead to the generation of visual imagery in dreams (Ogawa et al., 2005). RBD pathophysiology is also associated with damage to the visual stream, thereby contributing to the advancement of RBD symptoms (Ogawa et al., 2005).

**Figure 4:**
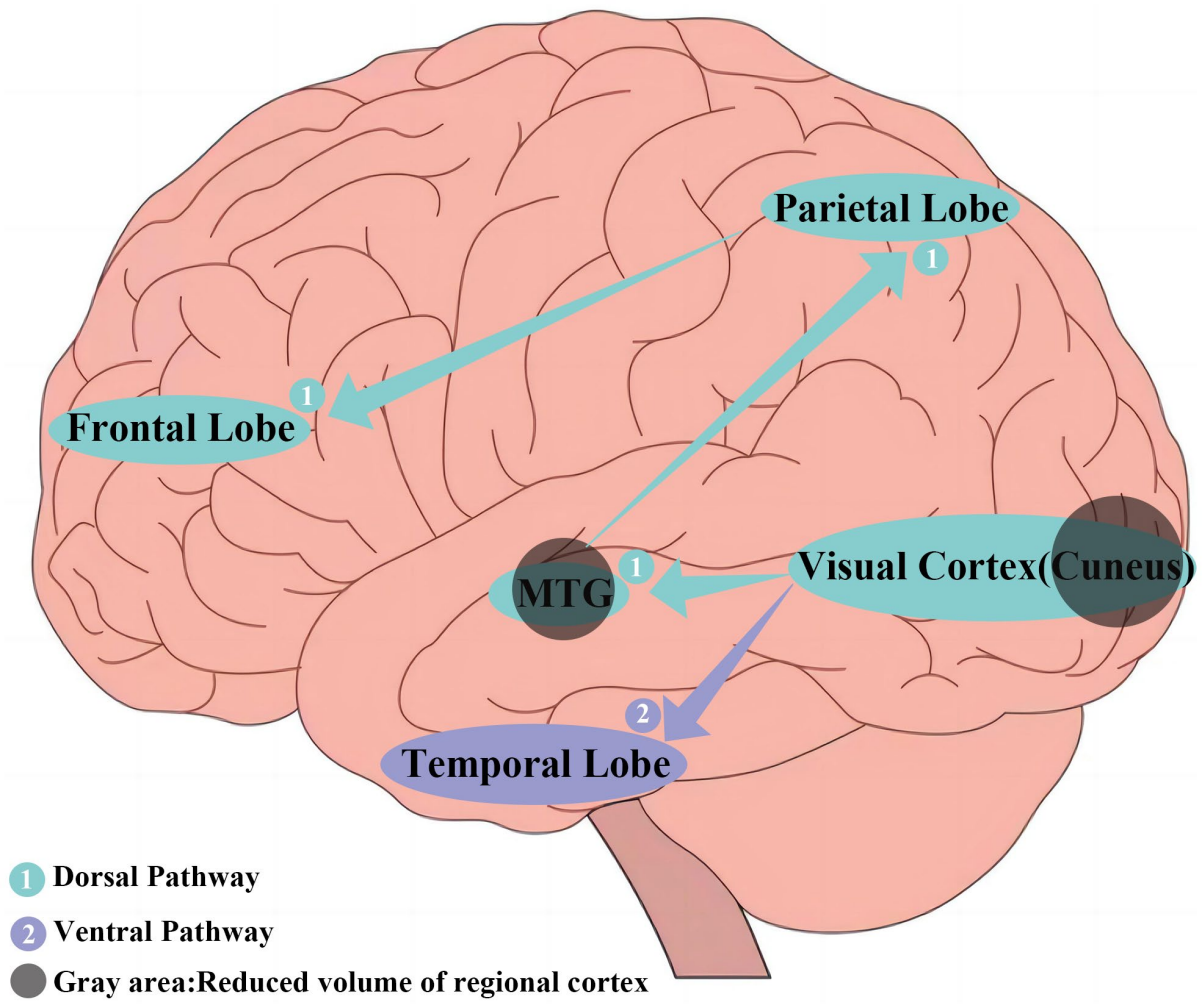
① The dorsal visual pathway radiating from the primary visual cortex to the middle temporal gyrus (MTG) and continues forward to the posterior parietal lobe and finally to the frontal lobe.② The ventral visual pathway running from the primary visual cortex (cuneus) to the inferior temporal lobe.

Additionally, we found lower FC between bilateral CUN and right MFG in PD-RBD patients. Visual information processing involves two distinct but complementary pathways: the ventral and dorsal visual pathways. The ventral pathway, from the primary visual cortex to the inferior temporal lobe, mediates visual information transformation into memory, recognition, and conscious perception. The middle temporal gyrus, a component of the occipital primary visual cortex, may affect visual information processing and related motor control to varying degrees due to atrophy. The dorsal pathway, from the primary visual cortex to the posterior parietal lobe and frontal lobe, is responsible for visual guidance and action generation (Milner, 2017) (Figure 4). The frontal cortex, a significant component of the motor cortex, has been implicated in dream-enacting behaviors in RBD (Rahayel et al., 2019; Sunwoo et al., 2019; Li et al., 2020). The observed lower FC between bilateral CUN and right MFG might be related to compromised sensorimotor integration in PD-RBD.

Several previous studies have explored the structural and functional alterations in RBD and PD, and our study is consistent with them, the decrease of GM volume in PD-RBD patients was mainly concentrated in posterior brain regions, occipital, temporal and parietal (Jiang et al., 2021; Chen et al., 2022; Donzuso et al., 2024). Based on these, using basic clinical characteristics, GMVs and FC values, we constructed ROC curves that demonstrated excellent predictive ability for PD with RBD. These results provide potential methods to reduce RBD attacks and improve PD’s prognosis.

## Limitations

Our study had some limitations. Firstly, although the RBDSQ used in this study showed good sensitivity/specificity during the diagnosis of RBD (Stiasny-Kolster et al., 2007), we did not include PSG, which is considered the gold standard for RBD diagnosis. Secondly, the sample size of this study was relatively small; besides, its cross-sectional design also limited causal interpretations. Additionally, our study focused only on GMV differences using VBM and did not investigate morphological parameters such as cortical thickness, which should be explored comprehensively in future studies. Our findings need to be validated and applicated in extensive longitudinal studies utilizing multiple imaging methods.

## Conclusion

In conclusion, our study revealed alterations in GMVs as well as FC among PD patients with RBD, which were closely linked to the severity of RBD. These findings might contribute to the identification of imaging markers for PD with RBD and the enhancement of our understanding of possible mechanisms.

## Data availability statement

The raw data supporting the conclusions of this article will be made available by the authors, without undue reservation.

## Ethics statement

The studies involving humans were approved by the Ethics Committee of the First Affiliated Hospital of Kunming Medical University (2019-L-46). The studies were conducted in accordance with the local legislation and institutional requirements. The participants provided their written informed consent to participate in this study.

## Author contributions

FW: Conceptualization, Data curation, Investigation, Methodology, Project administration, Writing – original draft. ZZ: Conceptualization, Data curation, Formal analysis, Methodology, Writing – review & editing. CZ: Data curation, Formal analysis, Investigation, Writing – review & editing. YoZ: Data curation, Formal analysis, Investigation, Writing – review & editing. YaZ: Data curation, Formal analysis, Supervision, Writing – review & editing. CL: Methodology, Software, Writing – review & editing. JC: Methodology, Software, Writing – review & editing. BL: Supervision, Validation, Visualization, Writing – review & editing. HR: Supervision, Validation, Writing – review & editing. XY: Conceptualization, Data curation, Resources, Supervision, Writing – review & editing.
